# Indirect meta-analysis comparing clinical outcomes of total cervical disc replacements with fusions for cervical degenerative disc disease

**DOI:** 10.1038/s41598-017-01865-3

**Published:** 2017-05-11

**Authors:** Bin Xu, Jian-xiong Ma, Jin-hui Tian, Long Ge, Xin-long Ma

**Affiliations:** 1 Biomechanics Laboratory of Orthopaedic Institute, Tianjin Hospital, Tianjin, China; 2 Evidence-based Medicine Center of Lanzhou University, Lanzhou, China; 3 Department of Orthopaedics, Tianjin Hospital, Tianjin, China

## Abstract

Anterior cervical discectomy and fusion (ACDF) and total cervical disc replacement (TDR) are considered effective treatments for patients with cervical degenerative disc disease (CDDD). An indirect meta-analysis including 19 randomized controlled trials (5343 patients) was conducted to compare the clinical outcomes of ACDF with TDR. Primary outcomes including functional indicators (NDI [neck disability index] score, neurological success and patient satisfaction), secondary outcomes including surgical outcomes (operation time, blood loss and length of stay) and secondary surgical procedures (secondary surgery at an adjacent level, secondary surgery at the index level, secondary surgery at both levels, removal, reoperation, revision and supplemental fixation) were included in the study. TDR using the Bryan disc was associated with a greater improvement in NDI score than ACDF (MD = −5.574, 95% CrIs [credible intervals] −11.73–−0.219). For neurological success, the Bryan (odds ratio [OR] = 0.559, 95% CrIs 0.323–0.955) and Prestige (OR = 0.474, 95% CrIs 0.319–0.700) discs were superior to ACDF. However, no differences in the patient satisfaction rate were shown between TDR and ACDF. For patients with CDDD, ACDF using allograft and a plate is most effective for determining the surgical parameters. Moreover, TDR using the ProDisc-C, Mobi-C, Prestige and Bryan discs are good choices for improving functional outcomes and reducing secondary surgeries.

## Introduction

Anterior cervical discectomy and fusion (ACDF) is considered the traditional standard operation and is widely performed to treat cervical degenerative disc disease (CDDD)^1, 2^. ACDF, which was described by Smith and Robinson^3^, may relieve pain significantly by improving nerve function and providing a stable biomechanical environment for the cervical operative segment^4^. However, approximately one-quarter of ACDF patients underwent reoperation procedures within ten years^5^.

Total cervical disc replacement (TDR) recently became an alternative to ACDF. TDR may provide normal structure and range of motion to the treated disc level. Moreover, adjacent segment disease is further reduced. However, TDR may lead to complications including heterotopic ossification, erosion of the replacements and increased flexibility of the adjacent cervical disc level.

To date, although several meta-analyses have compared the clinical effectiveness of total TDR and total ACDF, the outcomes were controversial. Whereas some results indicated that TDR was significantly superior to ACDF^6–8^ regarding several clinical outcomes, other results showed no significant difference between the two treatments^9–11^. Additionally, the clinical effectiveness among TDR using different replacements and ACDF using different implants remain unresolved.

The aim of this study was to perform an indirect meta-analysis to estimate the relative effectiveness of each type of TDR or ACDF using different implants on surgical parameters including operative time, blood loss and length of stay, functional indicators including the Neck Disability Index (NDI) score, Short Form 36 (SF-36) Physical Component Score (PCS), SF-36 Mental Component Score (MCS), Visual Analogue Scale (VAS) neck pain score, VAS arm pain score, neurological success, patient satisfaction, return-to-work status and secondary surgical procedures including secondary surgery at an adjacent level, both levels, the index level and removal, reoperation, revision, and supplemental fixation.

## Results

### Included studies

**Figure 1:**
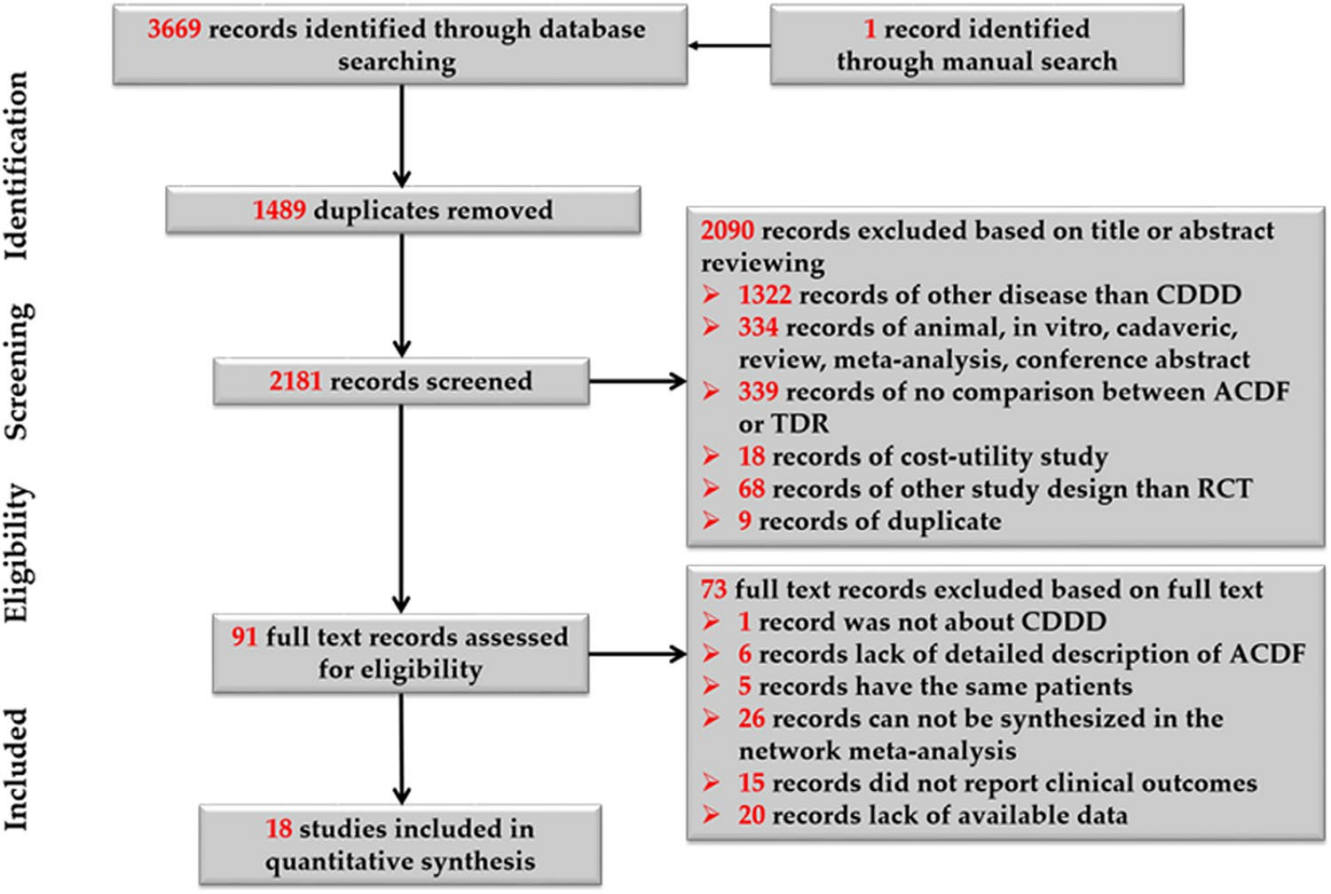
Flow chart of studies included in the meta-analysis.

Three thousand six hundred and sixty nine abstracts were retrieved from electronic searches. After duplicates were removed, the titles and abstracts of 2181 studies were screened. Finally, nineteen trials^12–30^ including twenty-four comparisons between TDRs and ACDF, were included in the review for data extraction and were included in the meta-analysis. The study selection process is shown in the PRISMA flow diagram (Fig. 1).

### Basic study characteristics

The characteristics of the included studies are shown in Table 1. In total, eight trials^12, 14, 18, 19, 25, 27–29^ compared the Bryan disc with ACDF using allograft bone and a plate, five trials^13, 22, 25, 26, 28^ compared the Prestige disc with ACDF using allograft bone and a plate, five trials^17, 23, 24, 28, 30^ compared the ProDisc-C disc with ACDF using allograft bone and a plate, three trials^16, 20, 21^ compared the Mobi-C disc with ACDF using allograft and a plate, and two trials^14, 15^ compared the Kineflex|C disc with ACDF using allograft bone and a plate.

**Table 1:** Basic characteristics of patients included in the meta-analysis.

Author(year)	country, search duration	intervention (TDR/ACDF)	No. of patients (TDR/ACDF)	Mean age(years), female(%)	Number of cervical levels	FDA IDE trial	Follow up (months)
Anderson P. A.^12^		Bryan disc/ACDF using allograft and a titanium alloy plate and screw	242/221		1 level	YES	36
Burkus J. K.^13^	October 2002 to August 2004	Prestige disc/ACDF using allograft with a plate	276/265	43.59, 53.80	1 level	YES	84
Coric D.^15^		Kineflex C disc/ACDF using allograft and a plate	136/133	43.80, 59.11	1 level	YES	24
Delamarter R. B.^17^	US, August 2003 to October 2004	ProDisc-C/ACDF using allograft and a plate	103/106	42.81, 54.55	1 level	YES	48
Garrido B. J.^18^		Bryan disc/arthrodesis using allograft and a plate	21/26	41.83, 36.16	1 level	NO	48
Heller J. G.^19^	May 2002 to October 2004	Bryan disc/ACDF using allograft and a plate	242/221	44.54, 45.19	1 level	YES	24
Hisey M. S.^20^	US, April 2006 to March 2008	Mobi-C disc/ACDF using allograft and a plate(SLIM-LOC^TM^/Sofamor Danek ATLANTIS^TM^/ATLANTIS^TM^ VISION anterior cervical plate system)	164/81	43.53, 53.47	1 level	YES	48
Mummaneni P.^22^	US, October 2002 to August 2004	Prestige disc/ACDF using allograft and a plate	276/265	43.59/53.80	1 level	YES	24
Murrey D. B.^23^	US, August 2003 to October 2004	Prodisc-C disc/ACDF using allograft with a plate	103/106	42.81, 54.55	1 level	YES	24
Murrey D. B.^24^	US, August 2003 to October 2004	Prodisc-C disc/ACDF using allograft with a plate	44/43	43.79, 44.83	1 level	YES	24
Riew K. D.^25^	US	Prestige ST disc/arthrodesis using allograft and a plate	59/52	44.62, 54.95	1 level	YES	24
	US	Bryan disk/arthrodesis using allograft and a plate	47/41	44.45, 62.50	1 level	YES	24
Riina j.^26^	US	Prestige ST disc/ACDF using allograft with a plate	10/9		1 level	YES	24
Sasso R. C.^27^		Bryan disc/ACDF using allograft and ATLANTIS VISION plate	56/59	44.35, 46.09	1 level	YES	24
Upadhyaya C. D.^28^	US,	Prestige ST/ACDF using allograft and a plate	253/220	43.58, 53.79	1 level	YES	24
	US,	Bryan Disc/ACDF using allograft and a plate	230/194	44.54, 51.94	1 level	YES	24
	US,	Prodisc-C disc/ACDF using allograft and a plate	101/100	42.80, 54.55	1 level	YES	24
Zhang X.^29^	China, May 2004 to May 2006	Bryan Disc/ACDF using allograft and a plate	60/60	45.17, 44.17	1 level	YES	24
Zigler J. E.^30^	August 2003 to October 2004	ProDisc-C/ACDF using allograft and a plate	103/106	42.81, 54.55	1 level	YES	60
Davis R. J.^16^	US, April 2006 to March 2008	Mobi-C disc/ACDF using allograft and a plate	225/105	45.59, 52.12	2 level	YES	48
Coric D.^14^		Bryan disc/ACDF using an allograft and a plate	21/41	not detailed	1 or 2 level	YES	24
		Kineflex C disc/ACDF using allograft and a plate	16/41	not detailed	1 or 2 level	YES	24
Jackson R. J.^21^		Mobi-C disc/ACDF using allograft and Slim-Loc Anterior Cervical Plate System/Sofamor Danek Atlantis or Atlantis Vision Anterior Cervical Plate System	179/81	not detailed	1 level	YES	60
		Mobi-C disc/ACDF using allograft and Slim-Loc Anterior Cervical Plate System/Sofamor Danek Atlantis or Atlantis Vision Anterior Cervical Plate System	234/105	not detailed	2 level	YES	60

FDA IDE trial: Food-and-Drug-Administration-regulated Investigational Device Exemption trial.

Seven studies^13, 17, 23, 25–27, 29^ reported NDI scores, nine studies^13, 17, 19, 22, 23, 25, 26, 28, 30^ reported neurological success rates, six studies^16, 17, 20, 24–26^ reported satisfaction rates, nine studies^16, 20, 23–27, 29, 30^ reported operation times, seven studies^16, 20, 23–25, 27, 30^ reported blood loss, six studies^16, 20, 23, 25, 27, 30^ reported length of stay, ten studies^12–15, 18, 19, 21, 22, 27, 29^ reported secondary surgery at an adjacent level, eight studies^12, 14, 15, 18–21, 27^ reported secondary surgery at the index level, seven studies^12, 14, 15, 18, 19, 21, 27^ reported secondary surgery at both levels, nine studies^13, 17–23, 25^ reported removal rates, fourteen studies^12–15, 17–23, 25, 27, 29^ reported reoperation rates, seven studies^13, 17, 19, 22, 23, 25, 27^ reported revision rates, and ten studies^13, 17–23, 25, 27^ reported supplemental fixation rates.

### Risk of bias

**Figure 2:**
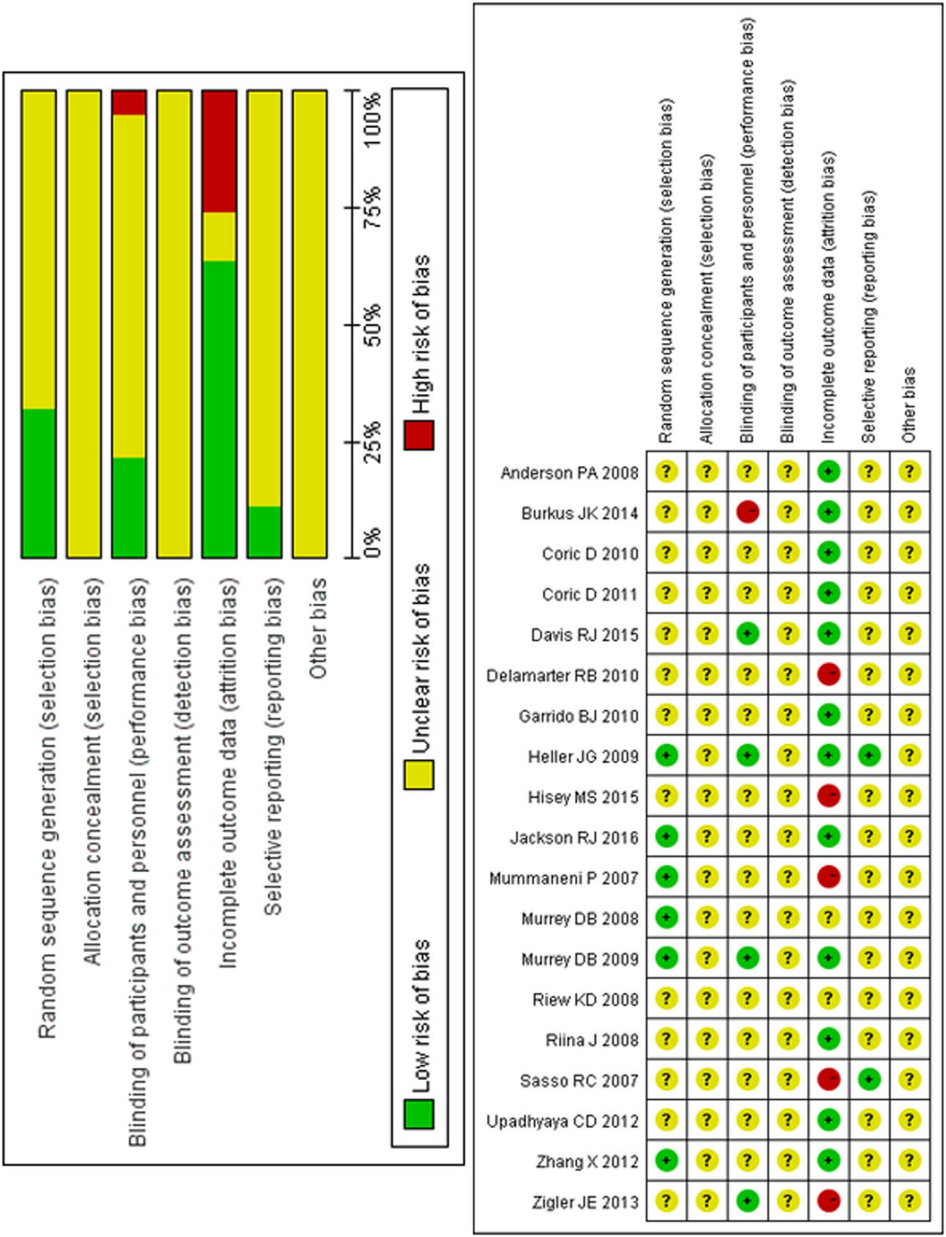
Risk of bias plot.

The risk of bias of the 18 studies is shown in Fig. 2. A total of six included studies had a low risk of bias for random sequence generation. None of the studies described allocation concealment. Four studies described the blinding participants and personnel. However, the participants and personnel were not blinded in one study. None of the included studies described blinding to outcome assessment. Twelve studies were considered at low risk for incomplete outcome data. Five studies were at high risk of incomplete outcome data. Only two studies were considered low risk for selective reporting.

### Assessments of heterogeneity

Heterogeneity for each outcome is shown in Table S1. For length of stay, high heterogeneity (I^2^ = 89.0%) was shown in the comparisons of the Bryan disc vs. ACDF using allograft and a plate. For NDI scores, moderate heterogeneity (I^2^ = 55.8%) was shown in the comparisons of Bryan disc vs. ACDF using allograft and a plate, indicating random variation between the investigations by chance. The remaining comparisons of TDR vs. ACDF showed minimal to low heterogeneity for all outcomes.

### Functional indicators

#### NDI score

**Figure 3:**
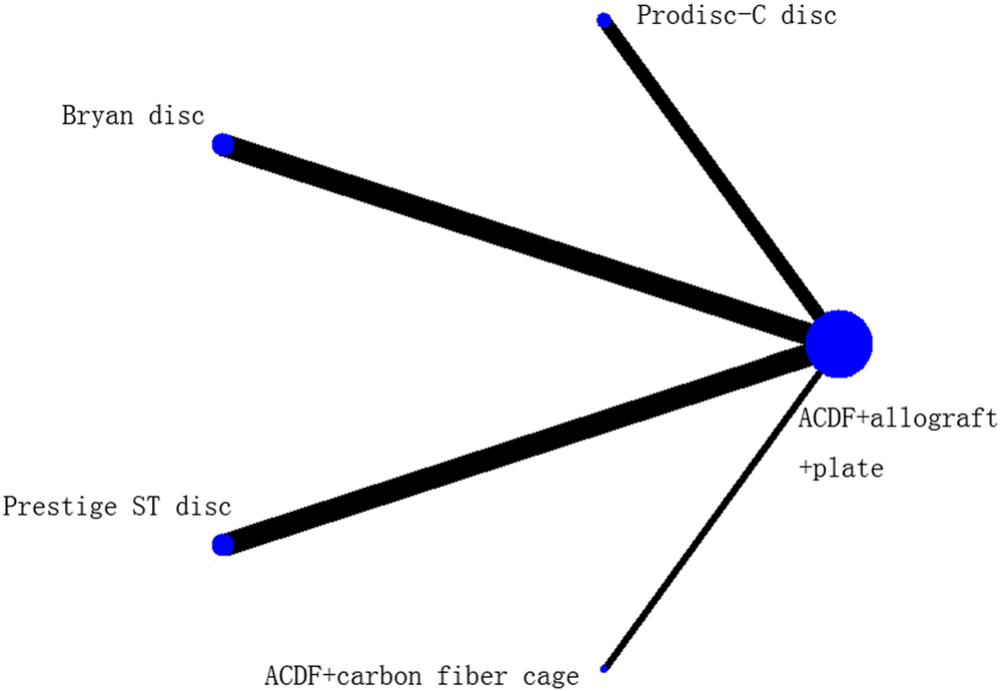
Network plot for NDI score.

The network plot of comparisons of NDI score is shown in Fig. 3. In total, 520 patients were assigned to ACDF using allograft and a plate, 166 patients to TDR using the ProDisc-C disc, 139 patients to TDR using the Bryan disc, and 280 patients to TDR using the Prestige ST disc.

Regarding NDI scores, the results showed that TDR with the Bryan disc was significantly more effective than ACDF using allograft bone and a plate (MD = −5.574, 95% CrI −11.73–−0.219) (Table 2).

**Table 2:** Indirect comparison results and SUCRA of NDI score.

**ACDF+ allograft+ plate** SUCRA = 0.2091	0.012 (−6.597 to 6.586)	−5.574 (−11.73 to −0.219)	−3.913 (−9.961 to 2.405)
NA	**ProDisc-C disc** SUCRA = 0.2391	−5.586 (−14.75 to 2.71)	−3.925 (−12.88 to 5.273)
NA	NA	**Bryan disc** SUCRA = 0.93476	1.66 (−6.351 to 10.62)
NA	NA	NA	**Prestige ST disc** SUCRA = 0.617

#### Neurological success

In total, 1406 patients were assigned to ACDF using allograft and a plate, 754 patients to TDR using the Prestige disc, 342 patients to TDR using the ProDisc-C disc, and 507 patients to TDR using the Bryan disc.

The current results indicated that TDR using the Prestige disc significantly improved neurological success than ACDF using allograft and a plate (OR = 0.474, 95% CrI 0.319–0.700). Moreover, TDR using the Bryan disc was significantly more efficacious in terms of neurological success than ACDF using allograft and a plate (OR = 0.559, 95% CrI 0.323–0.955) (Table S2).

#### Patient satisfaction

In all, 285 patients were assigned to ACDF using allograft and a plate, 58 patients to TDR using the Prestige disc, 109 patients to TDR using the ProDisc-C disc, and 338 patients to TDR using the Mobi-C disc.

The results showed no significant differences in the satisfaction rates between the pairwise comparisons (Table S3).

### Surgical Parameters

#### Operation time

In total, 547 patients were assigned to ACDF using allograft and a plate, 220 patients to TDR using the ProDisc-C disc, 338 patients to TDR using the Mobi-C disc, 68 patients to TDR using the Prestige ST disc, and 139 patients to TDR using the Bryan disc.

The patients who underwent ACDF using allograft and a plate experienced less operation time than those who underwent TDR using the ProDisc-C disc (MD = 8.368, 95% CrI 1.068–15.63), Mobi-C disc (MD = 16.97, 95% CrI 7.379–26.81), Prestige ST disc (MD = 14.52, 95% CrI 2.62–26.32) and Bryan disc (MD = 30.79, 95% CrI 22.79–39.23), respectively. Additionally, TDR using the Bryan disc was associated with significantly increased operation time than TDR using the ProDisc-C disc (MD = 22.42, 95% CrI 11.65–33.72), Mobi-C disc (MD = 13.82, 95% CrI 1.391–26.77) and Prestige ST disc (MD = 16.27, 95% CrI 1.94–31.18), respectively (Table S4).

#### Blood loss

In total, 435 patients were assigned to ACDF using allograft and a plate, 220 patients to TDR using the ProDisc-C disc, 338 patients to TDR using the Mobi-C disc, and 83 patients to TDR using the Bryan disc.

The meta-analysis results showed that ACDF using allograft and a plate was significantly associated with less blood loss than TDR using the ProDisc-C disc (MD = 21.81, 95% CrI 10.82–32.56) and Bryan disc (MD = 21.11, 95% CrI 4.333–37.29), respectively. Additionally, TDR using the Mobi-C disc was significantly associated with less blood loss than TDR using the ProDisc-C disc (MD = −23.38, 95% CrI −40.71–−5.79) and Bryan disc (MD = 22.69, 95% CrI 1.136–43.56) (Table S5).

#### Length of stay

In total, 392 patients were assigned to ACDF using allograft and a plate, 176 patients to TDR using the ProDisc-C disc, 338 patients to TDR using the Mobi-C disc, and 83 patients to TDR using the Bryan disc.

For length of stay, the results showed no significant differences between the pairwise comparisons (Table S6).

### Secondary surgical procedures

#### Secondary surgery at an adjacent level

In total, 1257 patients were assigned to ACDF using allograft and a plate, 433 patients to TDR using the Prestige disc, 603 patients to TDR using the Bryan disc, 365 patients to TDR using the Mobi-C disc, and 135 patients to TDR using the Kineflex|C disc.

ACDF using allograft and a plate showed a significantly higher rate of secondary surgery at an adjacent level than TDR with the Prestige disc (OR = 3.527, 95% CrI 1.396–9.439) and TDR with the Mobi-C disc (OR = 3.197, 95% CrI 1.185–8.908), respectively (Table S7).

#### Secondary surgery at the index level

In all, 886 patients were assigned to ACDF using allograft and a plate, 547 patients to TDR using the Bryan disc, 503 patients to TDR using the Mobi-C disc, and 135 patients to TDR using the Kineflex|C disc.

The results showed no significant difference between the pairwise comparisons in the rate for rate of secondary surgery at the index level (Table S8).

#### Secondary surgery at both levels

In total, 822 patients were assigned to ACDF using allograft and a plate, 547 patients to TDR using the Bryan disc, 365 patients to TDR using the Mobi-C disc, and 135 patients to TDR using the Kineflex|C disc.

ACDF using allograft and a plate showed a significantly higher rate of secondary surgery at both levels than TDR with the Mobi-C disc (OR = 3.155, 95% CrI 1–10.78) (Table S9).

#### Removal

In all, 1016 patients were assigned to ACDF using allograft and a plate, 492 patients to TDR using the Prestige disc, 166 patients to TDR using the ProDisc-C disc, 248 patients to TDR using the Bryan disc, and 503 patients to TDR using the Mobi-C disc.

For the removal rate, TDR using the ProDisc-C disc showed a significantly higher rate of removal surgery than when the Prestige disc was used (OR = 16.9, 95% CrI 1.027–803.6) (Table S10).

#### Reoperation

In sum, 1511 patients were assigned to ACDF using allograft and a plate, 433 patients to TDR using the Prestige disc, 166 patients to TDR the ProDisc-C disc, 650 patients to TDR using the Bryan disc, 503 patients to TDR using the Mobi-C disc, and 135 patients to TDR using the Kineflex|C disc.

Regarding the reoperation rate, TDR using the Mobi-C disc showed a significantly lower rate of reoperation surgery than ACDF using allograft and a plate (OR = 0.275, 95% CrI 0.103–0.740) (Table S11).

#### Revision

In all, 812 patients were assigned to ACDF using allograft and a plate, 492 patients to TDR using the Prestige disc, 166 patients to TDR using the ProDisc-C disc, and 266 patients to TDR using the Bryan disc.

The results indicated that TDR with the Prestige disc was significantly associated with a reduced rate of revision surgery than ACDF using allograft and a plate (OR = 0.077, 95% CrI 0.003–0.767). Additionally, TDR using the ProDisc-C disc was also significantly associated with a reduced rate of revision than ACDF using allograft and a plate (OR = 0.037, 95% CrI 0.000–0.629) (Table S12).

#### Supplemental fixation

In all, 1040 patients were assigned to ACDF using allograft and a plate, 433 patients to TDR using the Prestige disc, 166 patients to TDR using the ProDisc-C disc, 331 patients to TDR using the Bryan disc, and 503 patients to TDR using the Mobi-C disc.

TDR with the Prestige disc was significantly associated with a reduced rate of supplemental fixation surgery than ACDF using allograft and a plate (OR = 0.033, 95% CrI 0.001–0.305). Additionally, TDR with the Mobi-C disc was also significantly correlated with a reduced rate of supplemental fixation surgery than ACDF using allograft and a plate (OR = 0.116, 95% CrI 0.013–0.714) (Table S13).

#### Ranking of treatments

The ranking results of the treatments are shown in Tables 2 and S2 to S13. For reducing blood loss and operation time, ACDF using allograft and a plate may be the best choice. For reducing length of stay, secondary surgery rates at both levels and at the index level and the reoperation rate, TDR using the Mobi-C disc may be the best option. TDR using the Bryan disc for improving the NDI score and the Prestige disc for increasing neurological success, reducing secondary surgery at an adjacent level, removal, and supplemental fixation, and the ProDisc-C disc for improving satisfaction rate and reducing the revision rate were the best choices, respectively. ACDF ranked second in reducing length of stay but was second to last in reducing the removal rate. Whereas it ranked lowest in improving NDI, neurological success, patient satisfaction and reducing secondary surgery at an adjacent level, both levels and the index level, reoperation, revision and supplemental fixation.

#### Sensitivity analyses

The results of each outcome were not altered by the sensitivity analyses.

## Discussion

All relevant studies available concerning treatment of CDDD patients with TDR using the Prestige/ProDisc-C/Bryan/Mobi-C/Kineflex|C discs and ACDF using allograft and a plate were included in the present meta-analysis. In total, thirteen available outcomes were estimated in the indirect comparison using random-effects models. Six to fourteen studies were included for each outcome.

The ranking results showed that TDR with the Mobi-C disc may be the best choice to reduce the length of stay, secondary surgery rate at both levels, secondary surgery rate at the index level and reoperation rate. TDR using the Bryan disc may be the first choice to improve patient NDI scores. TDR using the Prestige disc may be the best choice to increase the neurological success rate and to reduce secondary surgery rate at an adjacent level, removal surgery rate and supplemental fixation rate. TDR with the ProDisc-C disc may optimally increase the satisfaction rate and reduce the revision rate. Interestingly, the ranking results indicated that ACDF using allograft and a plate may be the best choice only for reducing blood loss and the operation time. However, ACDF using allograft and a plate may be the worst choice for improving the NDI score, neurological success and the satisfaction rate, and for reducing the secondary surgery rate at an adjacent level, secondary surgery rate at both levels, secondary surgery at the index level, reoperation, revision and the supplemental fixation rate. For reducing the removal surgery rate, ACDF using allograft and a plate ranked second to last. That is, for patients with CDDD, TDR using replacements including the Mobi-C disc, Bryan disc, Prestige disc and ProDisc-C disc are better choices than ACDF using allograft and a plate.

The basic characteristics of the meta-analyses comparing TDR and ACDF are shown in Table S14. In total, sixteen published meta-analyses^6, 7, 9, 10, 31–42^ compared TDR using different replacements with ACDF. One published meta-analysis^11^ was a comparison study between TDR using the Bryan disc and ACDF. The last search date of the seventeen studies was from March 2011 to October 2015.

In all, five meta-analyses^32, 34, 35, 39, 41^ and the short-term results of one meta-analysis^11^ indicated that no significant difference existed between TDR and ACDF in improving the NDI score, although another five studies^7, 31, 38, 40, 42^ and the long-term results of one study^11^ showed that TDR was superior to ACDF, similar to the meta-analyses. The reasons for these findings may be as follows: (1) number of included studies differed significantly; (2) unfitted data was included in published meta-analysis; e.g., the data of Heller JG 2009^19^ included the NDI improvement from baseline but not NDI score at the last follow-up; (3) some outcome data lacked SD values such as those of Mummaneni P 2007 in Xing D 2013^39^, which were excluded from the current study; and (4) comparison of Nabhan A 2011 in Xing D 2013^39^ was between the ProDisc-C and ACDF using the Solis cage and a titanium plate, which was the only ACDF study included in the comparison.

For neurological success rate, twelve meta-analyses^6, 9, 31–34, 36–40, 42^ compared TDR and ACDF. Almost all results of the included studies were in accord with the current study except for Ren C 2014^37^, the short-term results of Boselie TF 2013^31^ and the midterm results of Zhang Y 2015^42^, perhaps because the numbers of patients included in those three studies were significantly less than the current study.

In total, two meta-analyses^7, 31^ compared TDR with ACDF in terms of patient satisfaction. Muheremu A 2015^7^ indicated that TDR was better than ACDF, which differed from the current study. The reasons for this findings may be that Heller JG 2009, Mummaneni P 2007 and Sun 2008 in Muheremu A 2015^7^ were not included in the current study. Data of the three studies were not identified in the articles.

In total, four meta-analyses^6, 9, 11, 32^ and five studies^6, 9, 11, 32, 33^ comparing TDR with ACDF investigated operation time reduction and length of stay separately. The results of these studies were in accord with the current study.

For blood loss, four meta-analyses^6, 9, 32, 33^ comparing TDR with ACDF and one meta-analysis^11^ comparing TDR using the Bryan disc with ACDF were published. The results of two studies^6, 9^ indicated no significant difference existed between TDR and ACDF, which differed from the current study. Two reasons may explain this difference: (1) several studies included in the published meta-analyses were excluded from the current study because only one study was included in the individual treatment comparison. For example, the comparisons of Philips FM 2015 and Coric D 2011 in Luo J 2015^6^ were TDR using the PCM disc vs. ACDF using a CSLP or SLIM LOC anterior plate and the Kineflex|C vs. ACDF using allograft and a plate, and (2) some outcome data lacked SD values such as those of Zhang X 2012 included in Rao M 2015^9^, which were excluded from the current study.

A total of thirteen meta-analyses^6, 9–11, 32–34, 37–42^ investigated the secondary surgery rate of TDR and ACDF. Almost all the meta-analyses results were in accord with the current study except for four studies^10, 38, 40, 42^. The results of secondary surgery at an adjacent level reported in two studies^10, 40^ and those of secondary surgery at the index level reported in three studies^38, 40, 42^ differed from the current study. The reason for these differences may be the significantly different number of patients included.

In all, two meta-analyses^33, 35^ described reoperation rates of TDR and ACDF. The results of Luo J^35^ showed that TDRs was associated with a greater reduction in the reoperation rate than ACDF, which was in accord with the current study. However, the results of Gao Y 2013^33^ showed that no significant difference existed between the two treatments, which differed from the current study. The only results on removal and supplemental fixation rates were also described in Gao Y 2013^33^, which also differed from the current meta-analysis. However, the article did not provide detailed outcome data about reoperation, removal and supplemental fixation. Therefore, the reason for the discrepancy between Gao Y 2013^33^ and the current study could not be discerned. Two studies^33, 41^ that compared TDR with ACDF investigated revision. The results of those studies were in accord with the current study.

The current meta-analysis has several strengths: (1) pairwise comparisons between ACDF using different implants and TDR using different replacements were conducted for the first time to provide comprehensive treatment information for patients with CDDD, and (2) available evidence was applied to the ranking of treatments to provide suggestions about the best choice for patients for each clinical outcomes. However, this article has some limitations: (1) this study lacks direct comparisons between TDR using different replacements and comparisons between different ACDF methods because only one study comparing outcomes between different TDRs was retrieved and less than 3 pairwise comparisons between different TDRs were included in the network plot for the outcome, (2) the numbers of patients included for each treatment were small, e.g., the sample sizes of the Bryan disc for blood loss and length of stay and the Prestige disc for operation time and satisfaction rate were less than 100; thus, the results presented in the current study should be interpreted cautiously, (3) different follow-up durations and races of patients in the included studies might be potential study confounders, (4) the results of outcomes including the SF-36 PCS, SF-36 MCS, VAS neck pain score, VAS arm pain score and return-to-work status were excluded from the current study and (5) the data of several studies were excluded because only one trial was included in each comparison. Therefore, the results of the current meta-analysis could not be assessed comprehensively.

## Conclusion

Available randomized controlled study data show that ACDF using allograft and a plate is superior to TDR in terms of surgical parameters, whereas TDR using the Bryan, Prestige, Mobi-C, and ProDisc-C discs are more effective than ACDF using allograft and a plate in regard to functional parameter improvement and secondary surgical procedure reduction. More head-to-head RCTs that directly compare ACDF using different implants or TDR using different replacements in a pairwise fashion are needed to comprehensively confirm the current results.

## Materials and Methods

### Inclusion criteria

#### Patients

Adult patients with symptomatic cervical degenerative disc disease including radiculopathy and/or myelopathy.

#### Interventions and comparisons

We included each pairwise comparison between total disc replacements (TDRs) using different replacements and anterior cervical discectomy and fusions (ACDFs) using different implants.

#### Outcomes

The primary outcomes included functional outcomes (NDI, SF-36 PCS, SF-36 MCS, VAS neck pain score, VAS arm pain score, neurological success, patient satisfaction and return-to-work status) and secondary outcomes included surgical parameters (operative time, blood loss and length of stay) and secondary surgical procedures (secondary surgery at an adjacent level, secondary surgery at the index level, secondary surgery at both levels, removal, reoperation, revision and supplemental fixation).

#### Study design

Randomized controlled trials were included in the study.

### Exclusion criteria

1. Patients with tumor, metabolic bone disease or trauma.
2. Lack of a detailed description of the surgical implants, e.g., TDR using the Bryan disc, ACDF using allograft and a plate.
3. Results of outcomes were not synthesized in the network meta-analysis in the following situations: (1) less than 3 different pairwise comparisons were included in the network plot for any outcome and (2) only one study was included in any pairwise comparison.

### Search and Selection of Studies

We searched CENTRAL, EMBASE and MEDLINE via Ovid SP on May 6, 2016 with no date/time, language, and document type limitations. Keywords were collected through expert opinion, literature review, controlled vocabulary (Medical Subject Headings = MeSH and Excerpta Medica Tree = EMTREE), and by reviewing the primary search results. Additionally, one study was hand searched through reference checking. The search strategies were developed with the assistance of a medical information specialist as reported in **Appendix 1**. Search results were de-duplicated in EndNote X5 and then sent to two authors for screening.

### Data extraction

Extraction of the available data was conducted by two reviewers (Xu B and Ma JX) separately. Any disagreement regarding the eligibility of data generated between the two reviewers was resolved via discussion among the investigators. Information concerning the author names, publication year, country, search duration, comparison of interventions, sample size, age and gender, number of treated levels, length of follow-up period, and clinical outcomes were extracted.

### Risk of bias assessment

The quality of the included studies was estimated using the Cochrane Collaboration tool^43^ for estimating risk of bias. Items for assessment included sequence generation, allocation concealment, blinding of outcome assessor, incomplete outcome data and selective outcome reporting. Each item was assessed using three degrees including high risk of bias, low risk of bias and unclear.

### Data synthesis and analysis

Each treatment effect was estimated using the mean difference (MD) with 95% credible intervals (CrIs) or odds ratio (OR) with 95% CrIs for continuous or dichotomous variables, respectively.

Statistical heterogeneity was estimated for each outcome of each pairwise comparison using I^2^ values. I^2^ values larger than 25%, 50% and 75% indicated low, moderate and high heterogeneity, respectively^44^.

A Bayesian indirect meta-analysis with random-effects models was conducted for each outcome using WinBUGS version 1.4 (UK). The model convergence was estimated using trace plots and the Brooks-Gelman-Rubin statistic; a burn-in of 10,000 followed by another 50,000 iterations was considered appropriate for convergence for each outcome^45^. The surface under the cumulative ranking area (SUCRA) was calculated to rank each treatment. SUCRA values of 100% and 0% indicate the best treatment and the worst treatment^46^, respectively.

Next, sensitivity analyses were conducted for each outcome by excluding each study included.

## Electronic supplementary material

Supplementary information
