# Supplementary material for: Indirect meta-analysis comparing clinical outcomes of total cervical disc replacements with fusions for cervical degenerative disc disease
Source: Sci Rep. 2017 May 11;7:1740. doi: 10.1038/s41598-017-01865-3 (PMC5431800; doi:10.1038/s41598-017-01865-3)
Supplement: Supplementary file 1 — Supplementary information [file 41598_2017_1865_MOESM1_ESM.pdf]

Indirect meta-analysis comparing clinical outcomes of total cervical disc replacements and fusions for cervical degenerative disc disease

Bin Xu[1], Jian-xiong Ma[1], Jin-hui Tian[2], Long Ge[2], Xin-long Ma[*, 3]

[1] Biomechanics Laboratory of Orthopaedic Institute, Tianjin Hospital, Tianjin, China.

[2] Evidence-based Medicine Center of Lanzhou University, Lanzhou, China.

[3] Department of Orthopaedics, Tianjin Hospital, Tianjin, China.

*Correspondence and requests for materials should be addressed to X-L.M. (email: maxinlong8686@sina.com)

S1 Table. Heterogeneity of the outcomes

	**Treatment 1**	**Treatment 2**	**Number of studies**	I^2	***P* value**
**NDI score**	ACDF+ allograft+ plate	ProDisc-C disc	2	0.0%	0.674
	ACDF+ allograft+ plate	Bryan disc	3	55.8%	0.104
	ACDF+ allograft+ plate	Prestige ST disc	3	0.0%	0.577
**neurological success**	ACDF+ allograft+ plate	Prestige ST disc	5	0.0%	0.983
	ACDF+ allograft+ plate	ProDisc-C disc	4	0.0%	0.593
	ACDF+ allograft+ plate	Bryan disc	3	0.0%	0.872
**satisfaction rate**	ACDF+ allograft+ plate	Prestige ST disc	2	0.0%	0.750
	ACDF+ allograft+ plate	ProDisc-C disc	2	0.0%	0.861
	ACDF+ allograft+ plate	Mobi-C disc	2	34.4%	0.217
**operation time**	ACDF+ allograft+ plate	ProDisc-C disc	3	0.0%	0.672
	ACDF+ allograft+ plate	Mobi-C disc	2	0.0%	0.404
	ACDF+ allograft+ plate	Prestige ST disc	2	20.3%	0.263
	ACDF+ allograft+ plate	Bryan disc	3	0.0%	0.427
**Blood loss**	ACDF+ allograft+ plate	ProDisc-C disc	3	0.0%	0.616
	ACDF+ allograft+ plate	Mobi-C disc	2	0.0%	0.890
	ACDF+ allograft+ plate	Bryan disc	2	0.0%	0.616
**length of stay**	ACDF+ allograft+ plate	ProDisc-C disc	2	0.0%	0.997
	ACDF+ allograft+ plate	Mobi-C disc	2	0.0%	0.410
	ACDF+ allograft+ plate	Bryan disc	2	89.0%	0.003
**secondary surgery at adjacent level**	ACDF+ allograft+ plate	Prestige ST disc	2	0.0%	1.000
	ACDF+ allograft+ plate	Bryan disc	6	0.0%	0.562
	ACDF+ allograft+ plate	Mobi-C disc	2	0.0%	0.691
	ACDF+ allograft+ plate	Kineflex|C disc	2	0.0%	0.982
**secondary surgery at**	ACDF+ allograft+ plate	Bryan disc	5	0.0%	0.549
	ACDF+ allograft+ plate	Mobi-C disc	3	0.0%	0.544

**index level**	ACDF+ allograft+ plate	Kineflex|C disc	2	0.0%	0.982
**secondary surgery at both level**	ACDF+ allograft+ plate	Bryan disc	5	0.0%	0.549
	ACDF+ allograft+ plate	Mobi-C disc	2	0.0%	0.691
	ACDF+ allograft+ plate	Kineflex|C disc	2	0.0%	0.982
**removal rate**	ACDF+ allograft+ plate	Prestige ST disc	3	0.0%	0.502
	ACDF+ allograft+ plate	ProDisc-C disc	2	0.0%	0.906
	ACDF+ allograft+ plate	Bryan disc	2	0.0%	0.638
	ACDF+ allograft+ plate	Mobi-C disc	3	0.0%	0.422
**reoperation rate**	ACDF+ allograft+ plate	Prestige ST disc	2	18.2%	0.269
	ACDF+ allograft+ plate	ProDisc-C disc	2	0.0%	0.904
	ACDF+ allograft+ plate	Bryan disc	7	0.0%	0.779
	ACDF+ allograft+ plate	Mobi-C disc	3	0.0%	0.439
	ACDF+ allograft+ plate	Kineflex|C disc	2	0.0%	0.868
**revision rate**	ACDF+ allograft+ plate	Prestige ST disc	3	0.0%	0.918
	ACDF+ allograft+ plate	ProDisc-C disc	2	0.0%	0.744
	ACDF+ allograft+ plate	Bryan disc	2	0.0%	0.369
**supplemental fixation rate**	ACDF+ allograft+ plate	Prestige ST disc	2	0.0%	0.937
	ACDF+ allograft+ plate	ProDisc-C disc	2	37.0%	0.208
	ACDF+ allograft+ plate	Bryan disc	4	0.0%	0.933
	ACDF+ allograft+ plate	Mobi-C disc	3	0.0%	0.829

S2 Table. Indirect comparison results and SUCRA of neurological success

ACDF+allograft+plate<br>SUCRA= 0.02465	0.474<br>(0.319 to 0.700)	0.658<br>(0.385 to 1.118)	0.559<br>(0.323 to 0.955)
NA	Prestige disc<br>SUCRA= 0.8458	1.383<br>(0.72 to 2.689)	1.181<br>(0.604 to 2.312)
NA	NA	ProDisc-C disc<br>SUCRA= 0.477	0.851<br>(0.402 to 1.809)
NA	NA	NA	Bryan disc<br>SUCRA= 0.6526

S3 Table. Indirect comparison results and SUCRA of satisfaction rate

ACDF+allograft+plate<br>SUCRA= 0.2613	1.161<br>(0.132 to 9.916)	0.458<br>(0.081 to 2.546)	0.489<br>(0.085 to 2.623)
NA	Prestige disc<br>SUCRA= 0.281	0.388<br>(0.027 to 6.308)	0.422<br>(0.027 to 6.303)
NA	NA	ProDisc-C disc<br>SUCRA= 0.7434	1.07<br>(0.088 to 11.71)
NA	NA	NA	Mobi-C disc<br>SUCRA= 0.7143

S4 Table. Indirect comparison results and SUCRA of operation time

ACDF+allograft+plate SUCRA= 0.98725	8.368 (1.068 to 15.63)	16.97 (7.379 to 26.81)	14.52 (2.62 to 26.32)	30.79 (22.79 to 39.23)
NA	ProDisc-C disc SUCRA= 0.6591	8.598 (-3.414 to 20.73)	6.148 (-7.727 to 20.17)	22.42 (11.65 to 33.72)
NA	NA	Mobi-C disc SUCRA= 0.4478	-2.451 (-17.85 to 12.65)	13.82 (1.391 to 26.77)
NA	NA	NA	Prestige ST disc SUCRA= 0.4011	16.27 (1.94 to 31.18)
NA	NA	NA	NA	Bryan disc SUCRA= 0.0047

S5 Table. Indirect comparison results and SUCRA of blood loss

ACDF+allograft+plate SUCRA= 0.7996	21.81 (10.82 to 32.56)	-1.573 (-14.89 to 11.92)	21.11 (4.333 to 37.29)
NA	ProDisc-C disc SUCRA= 0.1942	-23.38 (-40.71 to -5.79)	-0.692 (-20.61 to 18.93)
NA	NA	Mobi-C disc SUCRA= 0.8436	22.69 (1.136 to 43.56)
NA	NA	NA	Bryan disc SUCRA= 0.1626

S6 Table. Indirect comparison results and SUCRA of length of stay

ACDF+allograft+plate SUCRA=0.5497	0.103 (-0.824 to 1.032)	-0.079 (-1.031 to 0.824)	0.015 (-0.977 to 0.879)
NA	ProDisc-C disc SUCRA= 0.386	-0.182 (-1.529 to 1.11)	-0.089 (-1.454 to 1.154)
NA	NA	Mobi-C disc SUCRA= 0.6518	0.093 (-1.242 to 1.363)
NA	NA	NA	Bryan disc SUCRA= 0.4124

S7 Table. Indirect comparison results and SUCRA of secondary surgery at an adjacent level

ACDF+allograft+plate SUCRA= 0.116	3.527 (1.396 to 9.439)	1.785 (0.799 to 4.239)	3.197 (1.185 to 8.908)	1.204 (0.361 to 4.672)
NA	Prestige disc SUCRA= 0.8365	0.502 (0.145 to 1.844)	0.899 (0.227 to 3.623)	0.345 (0.071 to 1.701)
NA	NA	Bryan disc SUCRA= 0.4854	1.792 (0.471 to 6.41)	0.677 (0.153 to 3.131)
NA	NA	NA	Mobi-C disc SUCRA= 0.7818	0.376 (0.081 to 2.015)
NA	NA	NA	NA	Kineflex C disc SUCRA= 0.2802

S8 Table. Indirect comparison results and SUCRA of secondary surgery at the index level

ACDF+allograft+plate SUCRA= 0.1813	1.539 (0.630 to 4.044)	2.584 (0.988 to 6.405)	1.244 (0.333 to 5.334)
NA	Bryan disc SUCRA= 0.5534	1.668 (0.417 to 5.774)	0.811 (0.160 to 4.401)
NA	NA	Mobi-C disc SUCRA= 0.8607	0.488 (0.101 to 2.745)
NA	NA	NA	Kineflex C disc SUCRA= 0.4046

S9 Table. Indirect comparison results and SUCRA of secondary surgery at both levels

ACDF+allograft+plate SUCRA= 0.1812	1.548 (0.603 to 4.317)	3.155 (1 to 10.78)	1.27 (0.309 to 5.547)
NA	Bryan disc SUCRA= 0.5309	2.034 (0.450 to 9.51)	0.811 (0.143 to 4.836)
NA	NA	Mobi-C disc SUCRA= 0.896	0.400 (0.062 to 2.567)
NA	NA	NA	Kineflex C disc SUCRA= 0.3918

S10 Table. Indirect comparison results and SUCRA of removal rate

ACDF+allograft+plate SUCRA= 0.3395	3.243 (0.876 to 17.44)	0.196 (0.006 to 2.334)	1.342 (0.180 to 11.93)	2.923 (0.754 to 10)
NA	Prestige disc SUCRA= 0.8179	0.059 (0.001 to 0.973)	0.414 (0.031 to 5.143)	0.896 (0.100 to 5.144)
NA	NA	ProDisc-C disc SUCRA= 0.0682	7.201 (0.271 to 418.9)	14.64 (0.878 to 577.8)
NA	NA	NA	Bryan disc SUCRA= 0.4931	2.165 (0.163 to 22.08)
NA	NA	NA	NA	Mobi-C disc SUCRA= 0.7812

S11 Table. Indirect comparison results and SUCRA of reoperation rate

ACDF+allograft+plate SUCRA= 0.2059	2.144 (0.633 to 7.423)	2.842 (0.208 to 111)	1.455 (0.661 to 3.204)	3.638 (1.352 to 9.717)	0.947 (0.256 to 3.698)
NA	Prestige disc SUCRA= 0.6272	1.311 (0.076 to 57.91)	0.678 (0.157 to 2.858)	1.687 (0.348 to 8.124)	0.443 (0.074 to 2.753)
NA	NA	ProDisc-C disc SUCRA= 0.6393	0.514 (0.012 to 7.6)	1.263 (0.029 to 21.21)	0.326 (0.007 to 6.16)
NA	NA	NA	Bryan disc SUCRA= 0.4542	2.502 (0.712 to 8.731)	0.654 (0.141 to 3.171)

NA	NA	NA	NA	Mobi-C disc<br>SUCRA= 0.8402	0.262<br>(0.051 to 1.44)
NA	NA	NA	NA	NA	Kineflex C disc<br>SUCRA= 0.2332

S12 Table. Indirect comparison results and SUCRA of revision rate

ACDF+allograft+plate<br>SUCRA= 0.1712	12.94<br>(1.305 to 339.7)	27.21<br>(1.589 to 1854)	1.076<br>(0.030 to 37.35)
NA	Prestige disc<br>SUCRA= 0.7428	2.138<br>(0.026 to 248)	0.081<br>(0.001 to 5.589)
NA	NA	ProDisc-C disc<br>SUCRA= 0.8566	0.037<br>(0.000 to 3.886)
NA	NA	NA	Bryan disc<br>SUCRA= 0.2294

S13 Table. Indirect comparison results and SUCRA of supplemental fixation rate

ACDF+allograft+plate<br>SUCRA= 0.0624	30.71<br>(3.28 to 1347)	2.886<br>(0.212 to 57.49)	5.473<br>(0.866 to 55.95)	8.631<br>(1.401 to 75.25)
NA	Prestige disc<br>SUCRA= 0.8922	0.089<br>(0.001 to 4.086)	0.178<br>(0.003 to 4.299)	0.273<br>(0.004 to 6.454)
NA	NA	ProDisc-C disc<br>SUCRA= 0.3708	1.936<br>(0.062 to 62.07)	2.969<br>(0.097 to 89.4)
NA	NA	NA	Bryan disc<br>SUCRA= 0.5319	1.569<br>(0.085 to 25.81)
NA	NA	NA	NA	Mobi-C disc<br>SUCRA= 0.6428

S14 Table. Characteristics of meta-analyses published

Study	last search date	treatment /control	No. of patients (TDR/ACDF)	mean age TDR/ACDF (years)	Male /Female	treatment level	follow-up	outcomes
Yu L 2011	2011	Prestige ST(562), ProDisc-C(123), Bryan(319) /ACDF(972)	1004/972	/	/	1 level	2y,4y,5y	1. NDI at 24m: NSD (4 studies, 720 patients).<br>2. Secondary surgery at 24m: TDR＞ACDF (4 studies, 1328 patients).<br>3. Revision at 24m: TDR＞ACDF (3 studies, 865 patients).
Jiang H 2012	April,2011	Prestige ST(276), ProDisc-C(128), Bryan(316), PCM(151) /ACDF(874)	871/874	44.17/44.04	822/915	1 level	24m,36m	1. NDI at 24m: NSD (3 studies, 1046 patients).<br>2. Neurological success at 24m: TDR＞ACDF (3 studies, 1046 patients).<br>3. Secondary surgery at 24m at index level: NSD; 24m at adjacent level: TDR＞ACDF.(5 studies, 1334 patients)
McAfee PC 2012	/	Prestige(126), Bryan(229), ProDisc-C(99), PCM(188) /ACDF(549)	642/549	/	/	1 level	24m	Neurological success at 24m: TDR＞ACDF (4 studies, 1194 patients).
Yang B 2012	Sep,2011	Prestige ST(276), Bryan(263), Kineflex-C, Mobi-C, Advent(59), Kineflex-C(136) /ACDF(679)	734/679	/	559/585 (1 study unclear)	1 level<br>2 levels	48m,60m	Secondary surgery at 48m to 60m at adjacent level: NSD (3 studies, 1273).
Boselie TF 2013	July,2012	/	1262/1138	/	/	1 level	3m,1y,2y	1. NDI at 1y to 2y: TDR＞ACDF (6 studies, 1505 patients).<br>2. Neurological success at 3 months: NSD (1 study, 497 patients); 1y to 2y: TDR＞ACDF (3 studies, 1147 patients).<br>3. Patient satisfaction at 1y to 2y: NSD (2 studies, 498 patients).
Gao Y 2013	March,2011	Prestige(579), ProDisc-C(381), Bryan(746), PCM(423), not stated(100), Bryan, Kineflex-C, Discover(57), Prestige, Discover, Bryan, ProDisc-C(73), Kinefelx-	2445/2438	43.72/45.43 (2 studies unclear)	2345/2468 (2 studies unclear)	/	6m,12m, 18.2m, 24m, 37m, 48m,60m	1. Blood loss: TDR＜ACDF (3 studies, 575 patients).<br>2. Operation time: TDR＜ACDF (3 studies, 575 patients).<br>3. Neurological success at 24m,48m and 60m: TDR＞ACDF (4 studies, 1056 patients).<br>4. Secondary surgery at 12m and 24m:TDR＞ACDF(5 studies, 1237 patients).<br>5. Revision: TDR＞ACDF (unclear).

		C, Mobi-C, Advent(39), Bryan, Prestige(47) /ACDF(2438)						6. Reoperation: NSD (unclear).<br>7. Removal: NSD (unclear).<br>8. Supplemental fixation: TDR＞ACDF (unclear).
Xing D 2013	Jan,2012	Prestige(313), ProDisc-C(133), Bryan(242), Kineflex-C(136) /ACDF(793)	824/793	43.55/42.80	748/869	1 level	24m,36m, 48m	1. NDI at 24m and 48m: NSD (6 studies, 1374 patients).<br>2. Neurological success at 24m and 48m: TDR＞ACDF (5 studies, 1353 patients).<br>3. Secondary surgery at 24m and 48m: TDR＞ACDF（6 studies, 1539 patients）.
Yin S 2013	Jun,2012	Prestige ST(562), ProDisc-C(241), Bryan(585), Kineflex-C(136), PCM(272) /ACDF(1644)	1796/1644	43.70/44.21 (1 study unclear)	1375/1562 (2 studies unclear)	1,2,3 levels	1y,2y,3y, 4y,5y	1. NDI for short term: TDR＞ACDF (5 studies, 1171 patients); midterm: TDR＞ACDF (3 studies, 704 patients).<br>2. Neurological success for short term: TDR＞ACDF (5 studies, 1296 patients); midterm: TDR＞ACDF (3 studies, 703 patients).<br>3. Secondary surgery at short term at index level: TDR＞ACDF (5 studies, 1496 patients)；short term at adjacent level: TDR＞ACDF (5 studies, 1142 patients); midterm at index level: TDR＞ACDF (3 studies, 1213 patients); midterm at adjacent level: NSD (3 studies, 1213 patients).
Ren C 2014	2013	Prestige(276), ProDisc-C(103), Bryan(242), Bryan, Kineflex-C(41), Bryan, Kineflex-C, Prestige, ProDisc-C(173) /ACDF(722)	835/722	44.54/44.39 (1 study unclear)	674/785 (1 study unclear)	1 level<br>2 levels	48m,56m, 60m,72m	1. Neurological success at 48m and 60m: NSD (3 studies, 722 patients).<br>2. Secondary surgery at 48m,60 and 72m: TDR＞ACDF (4 studies, 1286 patients).
Gao F 2015	Oct,2014	/	2091/1934	43.29/43.88	1968/2057	1 level	24m,36m, 48m,60m	1. Blood loss at 24m, 60m: TDR＜ACDF (5 studies, 1247 patients).<br>2. Length of stay at 24m and 60m: NSD (6 studies, 1367 patients).<br>3. Operation time at 24m and 60m: TDR＜ACDF (6 studies, 1367 patients).<br>4. NDI at 24m: NSD (5 studies, 760 patients).<br>5. Neurological success at 24m and 60m: TDR＞ACDF (8 studies, 2220 patients).<br>6. Secondary surgery at 24m and 60m: TDR＞ACDF (6 studies, 2182 patients).
Luo J 2015	Jun,2014	/	884/834	43.50/44.02 (1 study unclear)	806/912	1 level	24m,36m, 48m,60m	Reoperation at 24m: TDR＞ACDF (4 studies, 1066 patients).

Luo J 2015a	April,2014	/	1672/1573	44.01/44.19	1553/1705	1 level	24m,36m, 48m,60m, 72m	1. Blood loss at 24m: NSD (3 studies, 881 patients).<br>2. Length of stay at 24m: NSD (4 studies, 1001 patients).<br>3. Operation time at 24m: TDR＜ACDF (4 studies, 1001 patients).<br>4. NDI at 24m: NSD (4 studies, 907 patients).<br>5. Neurological success at 24m: TDR＜ACDF (8 studies, 2428 patients).<br>6. Secondary surgery at 24m: TDR＞ACDF (6 studies, 2163 patients).
Muheremu A 2015	Jun,2013	/	/	/	/	1 level	1y,2y,3y, 4y,5y	1. NDI at 1y: TDR＞ACDF (11 studies, 1810 patients); 2y: TDR＞ACDF (11 studies, 2093 patients); 4y:TDR＞ACDF (4 studies, 837 patients).<br>2. Patient satisfaction at 1y: TDR＞ACDF (5 studies, 1276 patients); 2y: TDR＞ACDF (6 studies, 1767 patients); 4y: TDR＞ACDF (3 studies, 799 patients).
Rao M 2015	April,2014	/	2199/1975	44.14/44.47	2004/2169	/	2y,3y, 3.2y,4y, 5y	1. Blood loss at 2y and 5y: NSD (7 studies, 1529 patients).<br>2. Length of stay at 2y and 5y: NSD (6 studies, 1446 patients).<br>3. Operation time at 2y and 5y: TDR＜ACDF (7 studies, 1529 patients).<br>4. Neurological success at 2y, 3.2y, 4y and 5y: TDR＞ACDF (7 studies, 2313 patients).<br>5. Secondary surgery at 2y, 3y, 4y and 5y: TDR＞ACDF (14 studies, 2774 patients).
Wu AM 2015	Oct,2014	Prestige(212), ProDisc-C(72), Bryan(181), Bryan, Kineflex-C(41)<br>/ACDF(415)	506/415	43.95/44.46 (1 study unclear)	273/329 (1 study unclear)	1 level	4y,5y,7y	1. NDI at 4y and 7y: TDR＞ACDF (2 studies, 714 patients).<br>2. Neurological success at 4y and 7y: TDR＞ACDF (2 studies, 714 patients).<br>3. Secondary surgery at 4y, 5y and 7y at index level: TDR＞ACDF (4 studies, 921 patients); 4y, 5y and 7y at adjacent level: TDR＞ACDF (4 studies, 921 patients).
Zhang Y 2015	Dec,2014	Prestige(562), ProDisc-C(320), Bryan(585), Kineflex-C(136) PCM(189), Secure-C(151), Mobi-C(459), Discover(51), Bryan, Kineflex-C(41), Bryan, Kineflex-C or Discover(57)<br>/ACDF(2170)	2551/2170	43.94/44.26 (1 study unclear)	2108/2318 (1 study unclear)	1,2,3 levels	2y,3y,4y,5y	1. NDI at 2y: TDR＞ACDF (6 studies, 1280 patients); 4y and 5y: TDR＞ACDF (2 studies, 590 patients).<br>2. Neurological success at short term: TDR＞ACDF (8 studies, 2266 patients); midterm: NSD (2 studies, 589 patients).<br>3. Secondary surgery at short term at index level: TDR＞ACDF (5 studies, 1513 patients); short term at adjacent level: TDR＞ACDF (5 studies, 878 patients); midterm at index level: TDR＞ACDF (5 studies, 1651 patients) midterm at adjacent level: TDR＞ACDF(5 studies, 1651 patients).

Zhu Y 2015	Oct,2015	Bryan(925) /ACDF(891)	925/891	44.23/44.72 (1 study unclear)	888/903	1,2,3 levels	2y,3y,4y	1. Blood loss at 2y and 3y: NSD (3 studies, 318 patients).<br>2. Length of stay at 2y: NSD (2 studies, 235 patients).<br>3. Operation time at 2y and 3y: TDR＜ACDF (3 studies, 318 patients).<br>4. NDI at short term: NSD (2 studies, 533 patients); midterm: TDR＞ACDF (1 study, 319 patients).<br>5. Secondary surgery at short term: at index level: NSD (3 studies, 630 patients); short term at adjacent level: NSD (3 studies, 630 patients) midterm at index level: NSD (2 studies, 510 patients) midterm at adjacent level: NSD (2 studies, 510 patients).

Note: Results of outcomes were presented in the form of results (number of studies included, number of patients included).

NSD: No statistical difference between two treatments.

ACDF＞TDR: ACDFs were superior to TDRs on the outcome.

TDR＞ACDF: TDRs were superior to ACDFs on the outcome.

Appendix 1: Search Strategies

A. Cochrane Library

#1 (Bryan or Prestige or Discover disc Kineflex* or Prodisc* or Mobi or Mobi-c or PCM or porous coated motion):ti,ab,kw (Word variations have been searched)(261)

#2 ("Anterior Discectomy and Fusion" or "Anterior Discectomy with Fusion" or "Anterior diskectomy and Fusion" or "Anterior diskectomy with Fusion" or "Anterior Cervical Discectomy and Fusion" or "Anterior Cervical Discectomy with Fusion" or "Anterior Cervical diskectomy and Fusion" or "Anterior Cervical diskectomy with Fusion" or ACDF or "cervical arthroplasty"):ti,ab,kw (Word variations have been searched)(260)

#3 (disc or discs):ti,ab,kw (Word variations have been searched)(4241)

#4 MeSH descriptor: [Total Disc Replacement] explode all trees (68)

#5 MeSH descriptor: [Arthroplasty] explode all trees(4322)

#6 MeSH descriptor: [Diskectomy] this term only (450)

#7 MeSH descriptor: [Cervical Vertebrae] explode all trees(875)

#8 (prosthese* or articial or Replacement* or arthroplasty or "Cervical Vertebrae"):ti,ab,kw (Word variations have been searched)(21978)

#9 #4 or #5 or #6 or #7 or #8 (22530)

#10 #3 and #9 (800)

#11 #1 or #2 or #10 in Trials (Word variations have been searched)(843)

B. EMBASE

1 (Bryan or Prestige or Discover disc Kineflex$ or Prodisc$ or Mobi or Mobi-c or PCM or porous coated motion).ti,ab,kw. (6753)

2 ("Anterior Discectomy and Fusion" or "Anterior Discectomy with Fusion" or "Anterior diskectomy and Fusion" or "Anterior diskectomy with Fusion" or "Anterior Cervical Discectomy and Fusion" or "Anterior Cervical Discectomy with Fusion" or "Anterior Cervical diskectomy and Fusion" or "Anterior Cervical diskectomy with Fusion" or ACDF or "cervical arthroplasty").ti,ab,kw. (1863)

3 (disc or discs).ti,ab,kw. (79586)

4 exp total disc replacement/ or exp arthroplasty/ or intervertebral diskectomy/ or exp cervical spine/ or (prosthese$ or articial or Replacement$ or arthroplasty or Cervical Vertebrae).ti,ab,kw. (384109)

5 3 and 4 (9091)

6 or/1-2,5 (16166)

7 (clin$ adj2 trial).mp. (1106761)

8 ((singl$ or doubl$ or trebl$ or tripl$) adj (blind$ or mask$)).mp. (237920)

9 (random$ adj5 (assign$ or allocat$)).mp. (137471)

10 randomi$.mp. (856155)

11 crossover.mp. (75976)

12 exp randomized-controlled-trial/ (402622)

13 exp double-blind-procedure/ (130500)

14 exp crossover-procedure/ (46919)

15 exp single-blind-procedure/ (22010)

16 exp randomization/ (70405)

17 or/7-16 (1643873)

18 6 and 17 (1696)

C. MEDLINE

1 (Bryan or Prestige or Discover disc Kineflex$ or Prodisc$ or Mobi or Mobi-c or PCM or porous coated motion).ti,ab,kw. (5668)

2 ("Anterior Discectomy and Fusion" or "Anterior Discectomy with Fusion" or "Anterior diskectomy and Fusion" or "Anterior diskectomy with Fusion" or "Anterior Cervical Discectomy and Fusion" or "Anterior Cervical Discectomy with Fusion" or "Anterior Cervical diskectomy and Fusion" or "Anterior Cervical diskectomy with Fusion" or ACDF or "cervical arthroplasty").ti,ab,kw. (1343)

3 (disc or discs).ti,ab,kw. (62766)

4 exp Total Disc Replacement/ or exp Arthroplasty/ or Diskectomy/ or exp Cervical Vertebrae/ or (prosthese$ or articial or Replacement$ or arthroplasty or Cervical Vertebrae).ti,ab,kw. (307638)

5 3 and 4 (6328)

6 or/1-2,5 (12357)

7 exp clinical trial/ (735827)

8 exp randomized controlled trials/ (104687)

9 exp double-blind method/ (135160)

10 exp single-blind method/ (21774)

11 exp cross-over studies/ (38034)

12 randomized controlled trial.pt. (415252)

13 clinical trial.pt. (499795)

14 controlled clinical trial.pt. (90648)

15 (clinic$ adj2 trial).mp. (624358)

16 (random$ adj5 control$ adj5 trial$).mp. (563825)

17 (crossover or cross-over).mp. (76461)

18 ((singl$ or double$ or trebl$ or tripl$) adj (blind$ or mask$)).mp. (195100)

19 randomi$.mp. (672378)

20 (random$ adj5 (assign$ or allocat$ or assort$ or reciev$)).mp. (190248)

21 or/7-20 (1130617)

22 6 and 21 (1130)
